# Adaptive behaviour of the spinal cord in the transition from quiet stance to walking

**DOI:** 10.1186/1471-2202-13-80

**Published:** 2012-07-16

**Authors:** Mariano Serrao, Alberto Ranavolo, Ole Kæseler Andersen, Carmela Conte, Romildo Don, Francesca Cortese, Silvia Mari, Francesco Draicchio, Luca Padua, Giorgio Sandrini, Francesco Pierelli

**Affiliations:** 1 Department of Medical and Surgical Sciences and Biotechnologies, Sapienza University of Rome, Polo Pontino, Latina, Italy; 2 Rehabilitation Centre, Policlinico Italia, Rome, Italy; 3 Department of Occupational Medicine, INAIL, Rome, Italy; 4 Integrative Neuroscience group, Center for Sensory-Motor Interaction, Department of Health Science and Technology, Aalborg University, Aalborg, Denmark; 5 IRCCS “C. Mondino Institute of Neurology” Foundation, University of Pavia, Pavia, Italy; 6 Centro Auxologico, Milan, Italy; 7 Fondazione Don Gnocchi, Milan, Italy; 8 IRCCS Neuromed, Pozzilli, Italy

## Abstract

**Background:**

Modulation of nociceptive withdrawal reflex (NWR) excitability was evaluated during gait initiation in 10 healthy subjects to investigate how load- and movement-related joint inputs activate lower spinal centres in the transition from quiet stance to walking. A motion analysis system integrated with a surface EMG device was used to acquire kinematic, kinetic and EMG variables. Starting from a quiet stance, subjects were asked to walk forward, at their natural speed. The sural nerve was stimulated and EMG responses were recorded from major hip, knee and ankle muscles. Gait initiation was divided into four subphases based on centre of pressure and centre of mass behaviours, while joint displacements were used to categorise joint motion as flexion or extension. The reflex parameters were measured and compared between subphases and in relation to the joint kinematics.

**Results:**

The NWR was found to be subphase-dependent. NWR excitability was increased in the hip and knee flexor muscles of the starting leg, just prior to the occurrence of any movement, and in the knee flexor muscles of the same leg as soon as it was unloaded. The NWR was hip joint kinematics-dependent in a crossed manner. The excitability of the reflex was enhanced in the extensor muscles of the standing leg during the hip flexion of the starting leg, and in the hip flexors of the standing leg during the hip extension of the starting leg. No notable reflex modulation was observed in the ankle muscles.

**Conclusions:**

Our findings show that the NWR is modulated during the gait initiation phase. Leg unloading and hip joint motion are the main sources of the observed modulation and work in concert to prepare and assist the starting leg in the first step while supporting the contralateral leg, thereby possibly predisposing the lower limbs to the cyclical pattern of walking.

## Background

Despite considerable knowledge of the anatomical structures involved in gait initiation [1-6], the way in which descending commands from the brain activate the lower spinal centres in order to initiate the rhythmic alternating movements of walking remains poorly understood. Study of human gait initiation – a transition phase from quiet stance to steady-state walking – should make it possible to understand how neural rhythmic activity emerges in the spinal cord and spreads throughout it at multi-segmental level. Gait initiation comprises a preparatory and a stepping phase [7,8] in which the legs play different functional roles, serving to limit postural perturbation at the beginning of gait initiation [7,8] and subsequently to provide the necessary forward propulsive forces [8-10].

In this task, descending commands and load- and movement-related joint inputs converge on the spinal cord, probably interacting with the central pattern generator (CPG) to generate and control joint and muscle synchronisation during walking [11]. Knowledge of these interactions would improve understanding of how the muscle synergies essential to walking [12] are automatically selected at spinal cord level.

Investigation of spinal reflexes is an approach commonly used, in both animals and humans, to explore spinal behaviour during walking [13-15]. The modulation and integration of spinal reflexes during walking are crucial to the production of movement and also part of CPG organisation [16-18]. Among the spinal reflexes, the nociceptive withdrawal reflex (NWR) is easily evoked in many muscles of the arms and legs [19,20] and it is a useful tool for studying spinal cord function during limb movements in humans [21-26]. Although the NWR is not involved in voluntary movements, afferents belonging to this reflex do participate in movement through alternative excitatory and inhibitory spinal neural pathways [27,28].

Previous studies have demonstrated that the NWR is differently modulated in static stance vs steady-state cycling movement [29] and in symmetrical vs asymmetrical loading during stance [23]. We hypothesised that body unloading and joint kinematics mediate NWR modulation during gait initiation and that the flexion reflex modulation is more pronounced in the proximal than in the distal leg muscles, thereby enabling these muscles to assist the starting leg in the first step while supporting the contralateral leg. To test this hypothesis, we established the modulation pattern of major leg muscle responses following bilateral nociceptive sural nerve stimulation during gait initiation. Nerves were stimulated across different phases of the gait initiation task and different kinematic joint behaviours (e.g. flexion vs extension).

## Methods

## Participants

Ten healthy young men, aged 27–41 years, gave their written informed consent to participate in the study which had local ethics committee approval and complied with the Helsinki Declaration.

## Kinematic recordings

Gait analysis was performed using the SMART-D stereo-photogrammetric system (BTS, Milan, Italy) with eight infrared video-cameras for the acquisition of kinematic variables. Anthropometric data were collected for each subject according to Winter’s method [30].

Twenty-two retro-reflective spherical markers (15 mm in diameter) covered with reflective aluminum powder were placed over prominent anatomical landmarks in accordance with validated biomechanical models [31,32]. A calibration procedure was carried out prior to the first data capture. Kinematic data were acquired and digitised with a sampling rate of 120 Hz. Spatial accuracy was < 0.4 mm in the *x, y* and *z* dimensions.

## Kinetic recording

A sensorised multi-configuration walkway (sampling rate of 100 Hz ) incorporating two piezoelectric force plates (model 9286, Kistler Instruments, Winterthur, Switzerland) was used to acquire ground reaction forces. Plate positions within the acquisition volume were determined during the calibration procedure.

A single zone force-sensing resistor (model FSR 402, Interlink Electronics, Inc., Los Angeles, USA) made of thick polymer film (18.3 mm in diameter) was placed under the medial area of the forefoot of the starting leg in order to detect the toe-off moment.

## EMG recordings

Surface EMG signals were recorded using a sixteen-channel Wi-Fi PocketEMG system (BTS, Milan, Italy) operating at a sampling rate of 1000 Hz, and bandpass-filtered at 10–400 Hz.

EMG activity was recorded through a pair of Ag/AgCl surface electrodes (Medelec, Oxford, UK; diameter 1 cm, distance between the electrodes 2 cm), placed over the gluteus maximus (GMax), rectus femoris (RF), biceps femoris (BF), vastus medialis (VM), tibialis anterior (TA), and soleus (SOL) muscles of both legs, in accordance with European recommendations for surface electromyography [33]. A disposable reference electrode, embedded in an armband, was placed over the distal extremity of the right forearm.

## Stimulation protocol

In all subjects, the sural nerve was stimulated percutaneously using a pair of disposable surface Ag/AgCl electrodes (Medelec, Oxford, UK) applied immediately below and behind the lateral malleolus. The sural nerve of both sides was stimulated separately (in a random order).

The stimulus consisted of 25 ms trains of five rectangular pulses (1 ms duration, 200 Hz frequency) delivered through a constant current stimulator (Grass S-88 stimulator, Grass Medical Instruments, Quincy, Massachusetts USA) connected to the motion analysis system.

Pain threshold (PT) was assessed using a staircase method [34]. The intensity of the electrical shocks used for the electrophysiological measurements was adjusted to three times the PT (3xPT). The subjects were asked to score their pain perception after each stimulus on a 0–10 point visual analogue scale (VAS).

The electrical stimuli were delivered at intervals of at least 40 seconds to avoid reflex habituation.

## Procedure

Before starting formal measurements for the study, the subjects underwent an initial training session to familiarise them with the electrical stimulation procedure and pain intensity ratings and to reduce any effects due to arousal and/or anxiety.

The subjects stood upright, barefoot and motionless, on a force plate placed mid-way along an 8-metre walkway; they stood with their feet parallel and the inner edges of their heels approximately 20 cm apart. They were asked, as far as possible, to load each foot equally. From this upright standing position, the subjects were asked to walk forward at a comfortable speed, beginning with their preferred foot, as soon as they heard an acoustic signal and to stop at the end of the walkway. The distance between the two force plates was adjusted to ensure that the subjects, starting from the first platform, always took their first step onto the second platform. The acoustic signal was controlled by the experimenter and connected with the optoelectronic system. This procedure was performed repeatedly with one-minute rest periods after every five trials. We recorded a total of 120 trials per subject in all the participants. In 100 of the 120 trials, a single painful stimulus was delivered, in a random order (the same for each subject), to either the right (50 trials) or the left (50 trials) sural nerve (perturbed trials). The remaining 20 trials, performed without stimulus (unperturbed trials), were used as control conditions. The stimulus was delivered by the experimenter across the gait initiation task. In each subject, we also recorded 20 trials during quiet stance after sural nerve stimulation of both sides.

## Data analysis

### Kinematic and kinetic analysis (unperturbed trials)

To evaluate whole-body kinematics we calculated the instantaneous behaviour of the centre of mass (COM) and its maximum lateral displacement. The instantaneous behaviour of the COM was determined by means of whole-body gait analysis using a 15-segment model (head, thorax, 3-segment arms, pelvis, and 3-segment legs) [32].

To evaluate the segmental kinematics, the centres of rotation of the hip, knee and ankle joints were determined. Joint angles and angular velocities were calculated according to the model of Davis et al. [31]. The sign of the angular velocities was used as a parameter to categorise the joints as executing a flexion or an extension in the sagittal plane.

To evaluate whole-body kinetics, the instantaneous coordinates (anterior-posterior and mediolateral directions) of the centre of pressure (COP) were assessed. The COP, measured using the two force plates, was taken to correspond to the location of the ground reaction force vector.

The axes were defined in accordance with the standards of the International Society of Biomechanics (forward x and lateral z) [35].

### The phases of gait initiation

**Figure 1:**
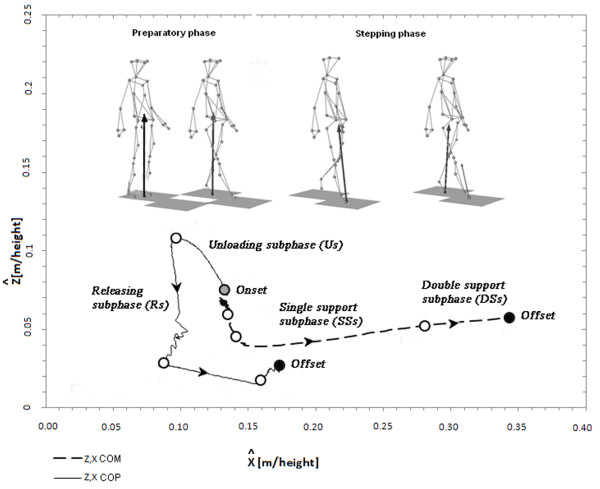
**Gait initiation phases.** Subdivision of gait initiation phases according to the COP and COM displacements in the medio-lateral and anterior-posterior directions: unloading (Us), releasing (Rs), single support (SSs) and double support (DSs) subphases.

On the basis of the behaviour of the COP and the COM, gait initiation was divided into two phases and four subphases [7,8], see Figure 1:

i. the *preparatory phase* (PP) was measured from the initial movement of the COP to the toe-off of the swing foot. This phase was further divided into two subphases, the release subphase (Rs) and the unloading subphase (Us). The onset of the Rs was taken to be the moment at which the COP moved posterolaterally towards the swing foot (displacement > 2 cm on both the x and y axes), while the end of the Rs corresponded to the moment at which the COP reached its maximum point before reversing direction. The onset of the Us was taken to be the point at which the COP, after reversing direction, moved towards the stance foot (displacement > 2 cm on both the x and z axes) and its end to be the moment at which the toe of the unloading swing foot was lifted from the ground, as shown by the signal coming from the sensor placed on the toe of the starting foot;

ii. the *stepping phase* (SP) was measured from the swing leg toe-off to the standing leg toe-off. This phase was further divided into two subphases: the single support subphase (SSs) and the double support subphase (DSs). The Ss was defined as the interval between swing limb toe-off and initial contact of the swing foot with the second force plate, and the DSSs as the interval between swing foot initial contact and toe-off of the opposite stance foot from the first force plate.

### EMG analysis (reflex detection)

In offline analysis the electrical stimulus artifact was detected and, for each muscle in each perturbed trial, the presence of the NWR in a time window of 60–200 ms (reflex window) from the stimulus artifact was investigated.

For the analysis of the quiet stance trials, we calculated the mean and SD values of the background EMG activity in the 140-ms time window preceding the stimulus delivery (control window) in all the perturbed trials. We took the mean EMG area of the control window as the baseline value and fixed this mean value +2SD as the threshold for detecting the presence/absence of the reflex in the reflex window (60–200 ms after the electrical stimulus).

The size of the NWR (δ_sd_EMG^post^), for the detected reflexes, was obtained using the following formula:

(1) $$\delta_{\text{sd}}{\text{EMG}}^{\text{post}}=\left({\text{EMG}}^{\text{post}}–{\text{EMG}}^{\text{pre}}\right)/{\text{EMG}}_{\text{SD}}{}^{\text{pre}}\text{,}$$

in which EMG^post^ is the muscle EMG area in the 60–200 ms window, EMG^pre^ is the baseline value, and EMG_SD_^pre^ is the standard deviation (SD) of the EMG baseline values. We used the SD of the baseline values as the denominator in the formula, in order to reduce variability due to different levels of EMG activity secondary to possible different degrees of muscle relaxation and signal noise.

For the analysis of gait initiation, the stimulus artifact was detected and analysed in relation to the corresponding subphase. The “reflex window” following the stimulus delivery (60–200 ms) in the perturbed trials was compared to the corresponding “control window” (140 ms) in the unperturbed trials in accordance with the method used previously in our laboratory [36,37]. In detail: i) the instant at which the stimulus was delivered in each perturbed trial was expressed as a percentage of the mean duration of the subject’s unperturbed trials; ii) the EMG area of each unperturbed trial was calculated in the corresponding time window; iii) the mean (and SD) EMG area of all the unperturbed trials was calculated and the mean value +2 SD was fixed as the EMG response threshold; v) the EMG reflex responses were considered present if the EMG area values of the perturbed trial exceeded this threshold.

The NWR size (δ_sd_EMG^p^) of the detected reflexes was obtained using the following formula:

(2) $$\delta_{\text{sd}}{\text{EMG}}^{\text{p}}=\left({\text{EMG}}^{\text{p}}–{\text{EMG}}^{\text{u}}\right)/{\text{EMG}}_{\text{SD}}{}^{\text{u}}\text{,}$$

in which EMG^p^ is the muscle EMG area in the single perturbed trial, EMG^u^ is the corresponding window in the unperturbed trial, and EMG_SD_^u^ is the SD of the EMG in the unperturbed trial window (control window). The SD of the baseline values was used to reduce the variability of the EMG background activity of the unperturbed trials during movement, thereby avoiding under- or overestimation of the NWR size.

All trials in which reflexes overlapped two consecutive phases were discarded from the analyses.

MATLAB software (Matlab 8.0, MathWorks, Natick, MA, USA) was used for the data processing.

### Repeatability of the kinematic variables

Repeatability of the hip, knee and ankle joint angles and angular velocities was investigated using the coefficient of multiple correlations (CMC). The CMC is a measure of the overall waveform similarity of a group of curves, and its magnitude is close to 1 if the waveforms are similar, or close to 0 if the lines are dissimilar. A CMC value > 0.7 indicates excellent repeatability [38].

Intrasubject repeatability was assessed by first computing the CMCs of each variable and subject over 100 unperturbed trials and then averaging these values over the 10 subjects. Intersubject repeatability was assessed by computing the CMCs for the unperturbed trials performed by all the subjects at the same time [38]. Furthermore, to test the possibility that motor behaviour may change due to the repetitive noxious stimuli, we also calculated the intrasubject CMCs from the mean curves calculated over groups of 10 consecutive unperturbed trials.

### Statistical analysis

The reflex probability rate was defined as the number of trials (expressed as a percentage) in which the reflex responses were present in a given muscle.

Chi-square/Fisher tests were used to compare the reflex probability rates, in each muscle, between gait initiation subphases (Rs, Us, SSs, DSs) and joint kinematic behaviours (i.e. flexion and extension). Chi-square/Fisher tests were also used to compare the reflex probability rate between homonymous muscles of the two legs in each single subphase.

To evaluate the effect of the subphases (a single within-subjects factor with four levels) on NWR size, one-way ANOVA for repeated measures was used after having performed the Kolmogorov-Smirnov test for normal distribution. A Greenhouse-Geisser correction was used when necessary to deal with violations of sphericity (i.e. inequalities in the variance of the differences between factors) [39]. The Bonferroni adjustment for multiple comparisons was used for pairwise post-hoc analyses.

A paired t-test was used to compare reflex size between joint flexion and extension.

**Figure 2:**
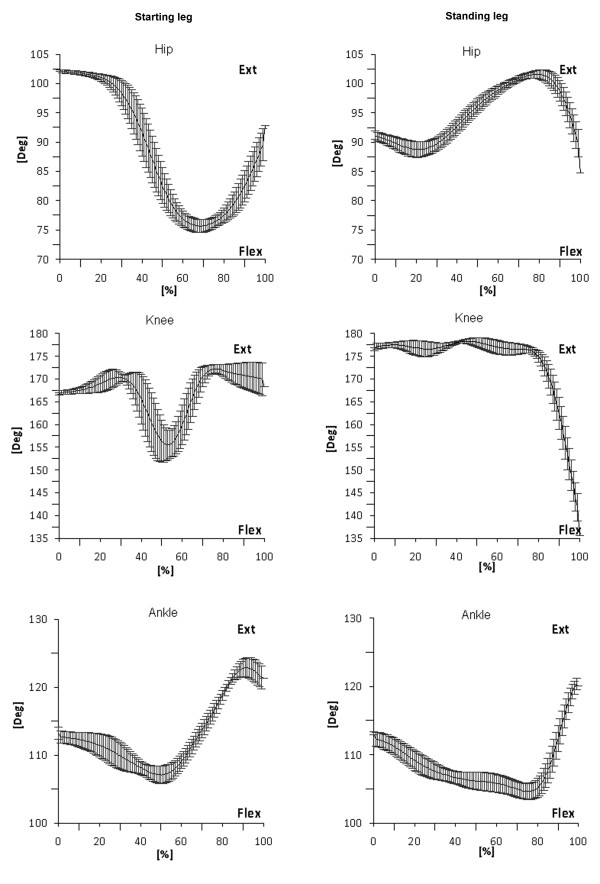
**Joint motion during gait initiation in starting and standing limbs.** The values on the horizontal axes are expressed as a percentage of the task duration.

Given the nature of the gait initiation task, only trials in which both flexion and extension movements were clearly observed in a given joint were included in the calculations (see Figure 2 for further details). Thus, flexion-extension movements of the hip, knee and ankle joints of the starting leg and of the hip of the standing leg were considered for the statistical analyses.

Pearson’s test was performed for each muscle to correlate NWR size with the corresponding unperturbed EMG background activity.

The non-parametric Friedman test was used to compare stimulation intensities and VAS scores between subphases.

**Figure 3:**
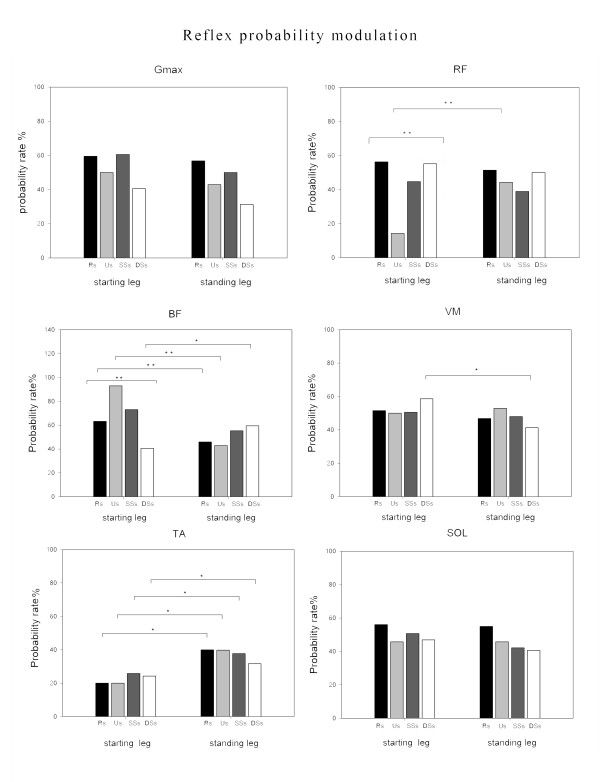
**Raw EMG data of the biceps femoralis muscle showing the NWR at the different phases of gait initiation in a representative subject.** Dotted lines indicate the stimulus delivery. Arrows indicate the reflexes. Note the higher size values in the unloading (Us) and single support (SSs) subphases.

All the analyses were performed using SPSS software (version 16.0). Descriptive statistics included reflex probability rates (expressed as <50%, 50-60%, 60-70% and >70%, see later in Figure 3), mean values and SD values. A p < 0.05 was considered statistically significant.

## Results

### Repeatability of the kinematic variables

Mean intrasubject and intersubject CMCs of the curves describing the kinematic variables were all > 0.88 and > 0.86, respectively. Intrasubject CMCs, calculated from the mean curves over groups of 10 consecutive unperturbed trials, were all > 0.82.

### Stimulation intensities and VAS scores

The mean stimulation intensities across subjects in the Rs, Us, SSs and DSs were 38.6 ±8.6 mA, 38.3 ±7.9 mA and 37.2 ± 11.0 mA and 38.1 ± 11.6 mA, respectively*.* The mean VAS scores in the Rs, Us, SSs and DSs were 6.8 ± 1.3, 6.6. ± 1.6 and 6.9 ± 2.2 and 6.7 ± 1.5, respectively. No significant differences in stimulation intensities and VAS scores were found between subphases (Friedman test, all, p > 0.05).

### Modulation of reflexes

A total of 392 perturbed trials for the starting leg and 390 trials for the standing leg were analysed during gait initiation; 218 trials were discarded from further analyses because the reflex windows overlapped two consecutive phases.

### Modulation related to subphases

**Figure 4:**
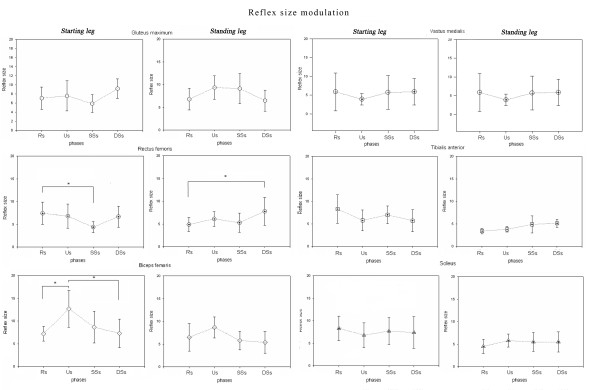
**Single**-**muscle reflex probability rates during different phases.** Group data of single-muscle reflex probability rates during different gait initiation phases in starting and standing legs. *Statistically significant differences between subphases.

**Figure 5:**
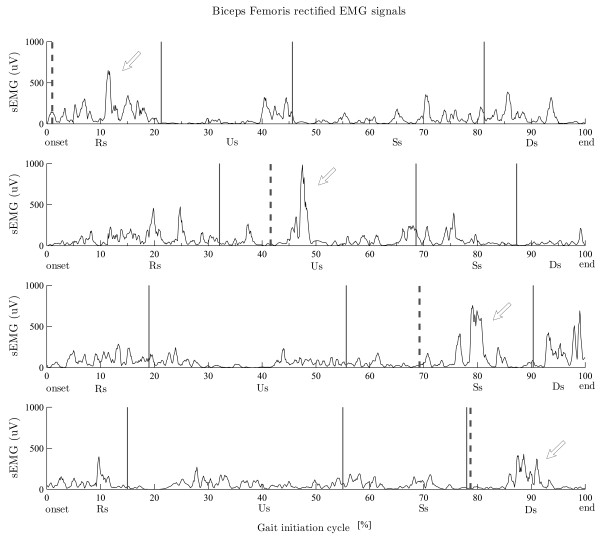
**Reflex size changes during different phases.** Reflex size changes in different muscles during gait initiation in starting and standing limbs. *Statistically significant differences between subphases at pairwise post-hoc analysis.

In the starting leg, both reflex probability and reflex size showed significant differences between subphases in the RF and BF muscles (Figures 4 and 5). In detail, in the RF muscle, the reflex probability and size values (r. size, one-way repeated ANOVA, main effect, F_(1.576,14.184)_ = 10.483, p = 0.003) showed the highest values in the Rs (Figures 4 and 5). Post-hoc analysis revealed significantly higher reflex size values in the Rs than in the SSs. In the BF muscle, both probability and size values (r. size, one-way repeated ANOVA, main effect, F_(3,27_ = 8.487, p = 0.002) showed the highest values in the Us (Figures 4 and 5). Post-hoc analysis revealed significantly higher reflex size values in the Us than in either the Rs or the DSs. No other differences were found for the other muscles across the subphases (all, p > 0.05).

Figure 4 gives raw EMG data for the biceps femoralis muscle of the starting leg showing the NWR at the different phases of gait initiation (in a representative subject).

In the standing leg, the reflex size (one-way repeated ANOVA, main effect, F_(3,27)_ =4 .634 p = 0.010) showed significant differences between subphases in the RF muscle, the values being higher in the DSs than in the Rs (Figure 5). No other differences were found in the other muscles across the subphases (all, p > 0.05).

### Modulation related to joint kinematics

In the starting leg, significant differences in reflex probability rate (Table 1) and reflex size, related to the hip joint kinematics of both legs, were observed in the RF and VM muscles. In the RF muscle, the reflex size values were higher during hip extension, compared to flexion, of the starting leg [r. size: 6.8 (1.5) vs 4.0 (1.0), paired t-test, t = −2.585, df = 9, p = 0.029] and both reflex probability and size values were higher during hip flexion, compared to extension, of the standing leg [r. size: 6.1 (1.1) vs 3.2 (0.4), paired t-test, t = 3.372, df = 9, p = 0.008]. In the VM muscle, both reflex probability rate and reflex size values were higher during hip joint extension than flexion of the starting limb [r. size: 5.2 (1.4) vs 10.6 (7.7), paired t-test, t = −2.372, df = 9, p = 0.049]. No other differences related to hip joint kinematics were found for the other muscles (all, p > 0.05).

**Table 1:** Differences in the reflex probability rate (%) in each muscle during flexion or extension of the ipsilateral and contralateral joints in both limbs (Chi-square p values)

	**Rest**	**Movement**											
		**Starting limb**											
Joints		Hip-i			Knee-i			Ankle-i			Hip-c		
Muscles		Flex	Ext	*p*	Flex	Ext	*p*	Flex	Ext	*p*	Flex	Ext	*p*
Gmax	88.0	56.1	42.9	0.267	56.0	52.6	0.704	61.5	46.0	0.780	58.8	47.5	0.200
RF	78.8	45.8	66.7	0.080	60.0	50.0	0.628	55.4	42.9	0.156	**58.8**	**37.3**	**0.015**
VM	90.0	**40.0**	**70.4**	**0.004**	49.1	43.5	0.512	47.0	55.1	0.164	45.3	45.5	0.988
BF	100	64.5	66.7	0.848	62.0	66.7	0.590	70.8	58.7	0.154	66.2	62.7	0.684
TA	94	8.4	19.0	0.140	14.0	7.7	0.249	7.7	12.7	0.249	8.8	11.9	0.573
SOL	100	48.6	61.9	.0284	53.5	50.0	0.740	48.6	53.6	0.642	46.2	54.9	0.411
		**Standing limb**											
Joints		Hip-i			Knee-c			Ankle-c			Hip-c		
Muscles		Flex	Ext	*p*	Flex	Ext	*p*	Flex	Ext	*p*	Flex	Ext	*p*
Gmax	90.0	46.4	46.8	0.945	36.0	52.6	0.067	47.7	44.4	0.712	**52.3**	**14.3**	**0.001**
RF	84.0	**50.0**	**32.2**	**0.043**	40.0	43.6	0.688	47.7	36.5	0.200	**38.3**	**61.9**	**0.045**
VM	90.0	20.5	27.8	0.423	23.6	26.0	0.764	20.0	25.8	0.540	23.8	26.7	0.765
BF	100	50.0	57.6	0.390	60.0	50.0	0.628	56.9	60.8	0.487	52.3	61.9	0.429
TA	94	26.5	39.0	0.133	42.0	25.6	0.053	27.7	36.5	0.287	35.5	14.3	0.057
SOL	100	52.9	40.7	0.167	40.4	51.3	0.224	53.8	39.7	0.108	**52.4**	**21.7**	**0.008**

*i = ipsilateral; c = contralateral; Gmax = gluteus maximus; RF = rectus femoris; VM = vastus medialis; BF = biceps femoris; TA = tibialis anterior; SOL = soleus.

In the standing leg, significant differences in both reflex probability (Table 1) and reflex size, related to hip joint kinematics of the starting leg, were found in the Gmax, RF, and SOL muscles. In particular, in the Gmax, the reflex showed higher values during hip joint flexion than extension [r. size: 8.2 (4.9) vs 3.2 (1.8), paired t-test, t = −2.771, df = 9, p = 0.022]. In the RF muscle, both reflex probability and reflex size were higher during the hip extension, compared to flexion, of the starting leg [9.1 (1.3) vs 5.2 (0.4), t = −4.014, df = 9, p = 0.003]. In the SOL muscle, both reflex probability and reflex size values were higher during hip flexion than extension of the starting limb (r. size: 6.5 (3.2) vs 3.2 (0.5), paired t-test, t = 3.731, df = 9, p = 0.005). No other differences related to hip joint kinematics were found for the other muscles (all, p > 0.05).

### Differences between homonymous muscles

**Figure 6:**
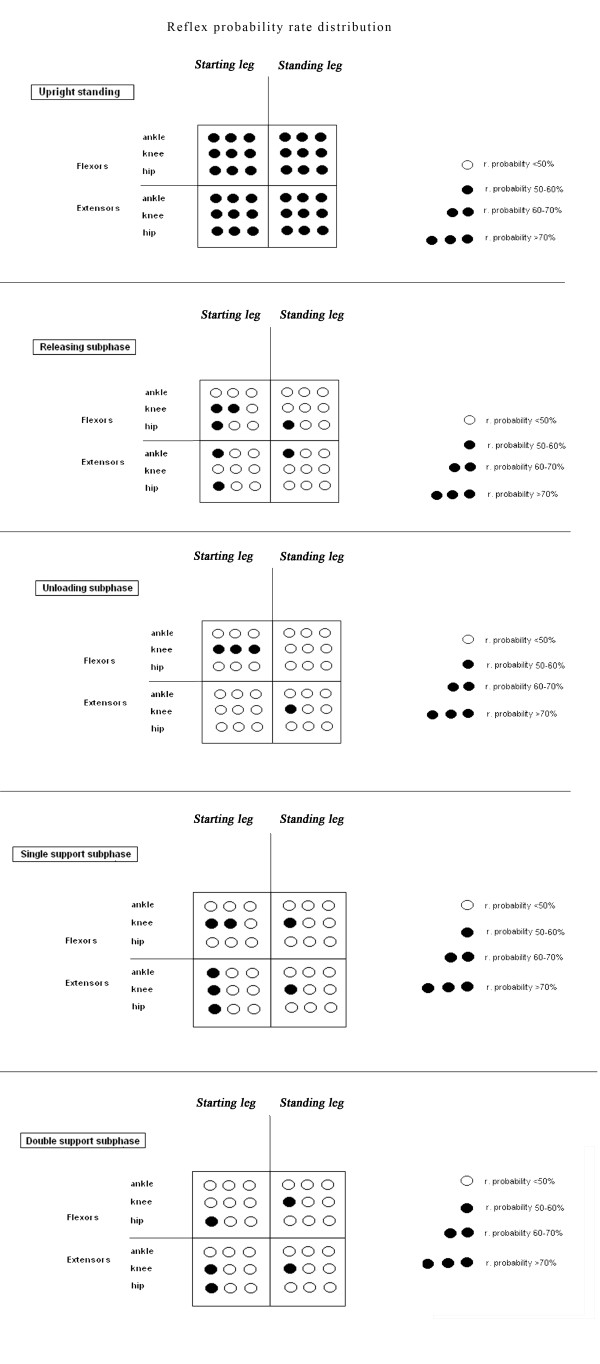
**Reflex probability distribution.** Distribution of muscle reflex probability rates during quiet upright stance and gait initiation.

Significant differences in reflex probability rates were found when considering the homonymous RF, BF, VM and TA muscles. In the RF muscle, the reflex values were lower in the starting (probability rate: 18%) than in the standing leg (probability rate: 48%) during the Us (Figures 4 and 6). In the BF muscle, the reflex values during the Rs and Us were higher in the starting (probability rate: 64% and 95%) than in the standing leg (probability rate: 47% and 43%) during the Rs and Us, respectively, and lower during the DSs (probability rate: 42% in the starting leg vs 59% in the standing leg) (Figures 4 and 6). In the VM, the reflex values were higher in the starting (probability rate: 58%) than in the standing leg (probability rate: 42%) during the DDs (Figures 4 and 6). In the TA muscle, the reflex probability rate was lower in the starting than in the standing leg across all the subphases (Figure 4).

### Association between background EMG and reflex size

No correlation was found between reflex size and background EMG activity during movement in any muscle (all, p > 0.05).

## Discussion

In the present study, we examined multi-muscle and multi-joint NWR modulation during the transition from quiet stance to walking.

The main results can be summarised as follows: i) the NWR is phase-dependent. The excitability of the reflex (in terms of probability rate and size) is increased in the hip and knee flexor muscles of the starting leg during the first subphase (Rs), i.e. just prior to the occurrence of any movement, and in the ipsilateral knee flexor muscles as soon as the leg is unloaded (Us); ii) the NWR is hip joint kinematics-dependent in a crossed manner, i.e. the excitability of the reflex is enhanced in extensor muscles of the standing leg during hip flexion of the contralateral leg, whereas it is enhanced in the hip flexors of the standing leg during hip extension of the contralateral leg.

### NWR modulation related to subphases

The NWR probability rate and size showed significant subphase-related modulation in the BF and RF muscles of the starting leg and in the RF muscle of the standing leg. Furthermore, significant differences in the reflex probability rate were found between the homonymous muscles of the two legs in almost all the subphases.

During the first gait initiation subphase (RSs), bodyweight was transiently shifted towards the starting leg as shown by the early COP displacement (Figure 1). The movement of the COP towards the starting leg has been suggested to be an anticipatory mechanism serving to stabilise the body before the postural perturbation [40]. Interestingly, the reflex probability rate and size in the RF muscle were higher in this subphase than in the others, while no significant differences were found in the same muscle of the standing leg (Figures 4 and 5). Furthermore, the reflex probability rate was significantly higher in the BF muscle of the starting leg than in that of the standing leg in the RSs. These results suggest that the descending pathways conveying the commands for the motor programming of gait initiation activate the spinal circuitries mediating the NWR by enhancing the withdrawal reflexes in the hip flexors (taking into account the bi-articular function of the RF muscle), as well in the knee flexors of the starting leg (Figure 6). This activation, which may serve to prepare and assist the leg in the first step, takes place just before any movement actually occurs, even though the COP shifts towards the starting foot, suggesting that the intentional descending commands interact with the spinal circuitry in accordance with the role that the CNS assigns to each leg (starting or standing) at the very beginning of gait.

A main finding of our study was that the NWR was clearly subphase-dependent in the BF muscle of the starting leg (Figures 4,5 and 6), the reflex values being higher in the unloading subphase (Us) compared to the other subphases. In the Us, the COP moved towards the standing leg (Figure 1), thus quickly unloading the starting leg during this double support configuration. This clearly indicates that the sudden unloading of the leg is the main event bringing about the increase of the withdrawal reflex in the knee flexor muscles. It is known that in lower mammals the response to loading is related to locomotor circuits in the spinal cord [41,42]. There is evidence that polysynaptic cutaneous reflexes (both painful and non-painful) are load-dependent. Indeed, the excitability of cutaneous reflexes is modified in symmetrical stance, compared to seated or prone postures [43,44], as well as in asymmetrical stance in the loaded compared to the unloaded leg [23]. A rich vein of research has revealed a phase-dependence (swing versus stance) of noxious and non-noxious cutaneous reflexes aimed at achieving leg withdrawal or maintenance of balance in the context of the gait cycle (for review see 15 and 45). This modulation of the reflex may reflect, at least in part, the effects of leg loading-unloading on spinal cord neuron excitability during the gait cycle.

The reversal of behaviour, in terms of NWR probability rate, seen in the knee flexor (BF) compared to the knee extensor (VM) muscles of the two legs during the DSs (Figures 4 and 6), when the leg is reloaded, further strengthens the idea that the shift of bodyweight from one leg to the other is the main factor in bringing about the increase/decrease in reflex activity at knee joint level. This finding is also in line with previous reports, in humans, of a facilitation of the cutaneous reflexes in knee extensors (i.e. the VL) [45,46] during the swing-to-stance phase, a finding which suggests that this is a safety mechanism serving to increase knee stiffness and reduce the possibility of leg collapse [45].

Surprisingly, no significant modulation of the reflex between subphases was found in the ankle muscles. Furthermore, the reflex probability rate in the TA muscle of the starting leg was low throughout the gait initiation task (Figure 3). Ankle muscles are known to be highly responsive to loading-unloading conditions [47,48]. One possible explanation for this could be that these muscles are under wide supraspinal control during early gait initiation. Indeed, there is growing evidence that the corticospinal control over distal muscles might be stronger than in proximal muscles during walking [49,50]. This descending control might be even more pronounced during gait initiation. In particular, the EMG activity is typically suppressed in the SOL muscle and enhanced in the TA muscle during gait initiation [51]. This suggests that voluntary recruitment of motoneurons prevails over the reflex response in these muscles, most likely in order to avoid mechanical perturbations and instability. Indeed, McIlroy et al. (1999) and Bent et al. (2001) [52,53] showed that, during the anticipatory postural adjustments (equal to the early part of gait initiation), the CNS is able to anticipate the shift of the COP in the mediolateral direction and to delay the occurrence of withdrawal reflexes in order to preserve balance.

### Modulation related to joint kinematics

The present study provides evidence that, irrespective of subphase-related modulation, dynamic joint motion regulates reflex variation among muscles in both legs.

Hip joint flexion of the starting leg led to an increase in the reflex probability rate and/or size in the Gmax, RF and SOL muscles of the standing leg (hip, knee and ankle extensors, respectively). This finding is interpretable as a reflex-mediated multi-joint extensor synergy serving to preserve the balance of the standing leg. Conversely, hip joint extension of the starting leg led to an increase in the reflex probability rate and/or size in the RF muscle of the standing leg (hip flexor) and in the RF and VM muscles (knee extensors) of the starting leg. The first of these findings may be interpreted as an enhanced flexion response serving to prepare for and support the first step of the contralateral leg (at the end of the stance phase), by helping to initiate the swing phase; while the latter finding may constitute an enhanced extensor response helping the ipsilateral leg (at the end of the swing) to accept the bodyweight at early stance.

Hip joint flexion of the standing leg induced increased reflex activity only in the RF muscle (knee extensor) of the starting leg, probably serving to support the knee extensors when the starting leg makes contact with the ground.

From these results, the RF muscle certainly seems to be extensively modulated by both bodyweight shift and joint motion in accordance with its bi-articular function (hip flexor or knee extensor). This bi-articular function of the RF muscle is typically observed during steady-state walking [40].

The limited reflex modulation induced by hip joint motion of the standing leg may reflect the limited movement performed by the standing leg during the gait initiation task (Figure 2). These results suggest that hip proprioceptive inputs are important signals for controlling locomotor activities once the legs start moving, as revealed during fictive locomotion in spinal animals [54,55].

### Neural mechanisms

Different afferent fibres, called flexor reflex afferents, may evoke a flexion reflex in both humans and animals [56-58]. Included in this group of afferents are cutaneous low-threshold mechanoreceptors, cutaneous nociceptive afferents, group II, III and IV muscle afferents, and joint afferents. In the present study, modulation of the NWR in the hip and knee muscles was clearly related to bodyweight shift and hip joint motion. Although a convergence from many afferent sources may be hypothesised (including inputs from labyrinth and neck receptors), group II ankle muscle afferents, cutaneous afferents conveying inputs from the plantar foot mechanoreceptors [59,60], and hip joint afferents [61] may all play key roles in modulating the NWR during gait initiation. In particular, group II afferents from ankle muscles have been demonstrated to play an important role in the control of bipedal stance and gait [62]. It has been shown that stimulation of the gastrocnemius medialis and tibialis anterior nerves evokes a group II-mediated EMG facilitation in thigh muscles, with enhanced responses while leaning forwards or backwards [62]. During gait initiation, when the body leans forwards, the ankle muscles contract while stretched, and thus it is likely that there is strong discharge from group II fibres which could facilitate the NWR in the proximal muscles. Furthermore, sensory information coming from plantar cutaneous afferents appears to play an important role in regulating stepping during human gait, facilitating control of compensatory stepping reactions. In particular, mechanoreceptors responding to pressure on the sole of the foot may be involved in sensing and controlling heel contact and subsequent weight transfer during termination of forward steps, and in maintaining stability during the prolonged swing phase of lateral crossover steps [63].

In our study, the afferents from the hip joints seem to be determinant in modulating the NWR during gait initiation. Our findings are in line with previous studies documenting that hip position entrains the activity of flexors and extensors during fictive locomotion in spinal animals [54,55]. In spinal cord injured humans, hip flexion and extension movements either suppress or enhance the excitability of the flexor reflex pathways [64-67], suggesting that the inputs from the hip region are fully integrated by interneuronal circuits associated with motor control.

### Functional considerations

The flexion withdrawal reflex in mammals is believed to incorporate interneuronal circuits that contain elements of the stepping generator (i.e. the CPG) [68,69]. However, in humans, despite the finding of stepping in anencephalic infants [70] and of alternating flexor-extensor bursts in patients with spinal cord lesions [71,72], the existence of a spinal CPG has been hypothesised on the basis of extrapolations from simpler, animal models [11,73].

Our findings suggest that in gait initiation NWR modulation and, possibly, recruitment of the CPG in the starting leg follows a well-ordered sequence: descending inputs, leg unloading, hip joint motion. Thus, after selective and asymmetrical excitation of the spinal substrate mediating the NWR (hip and knee flexor muscles of the starting leg) by descending motor commands, leg unloading and hip joint motion work in concert to produce an alternating (right and left) and crossed (flexors and extensors) activation mainly of the hip and knee joint muscles, predisposing the legs to the cyclical pattern of steady-state walking.

Herein, we speculate that CPG activation does not occur statically, in advance of the joint motion and torque changes, but rather that it emerges dynamically through the movement and unloading themselves. Conceptually, the CPG could be viewed as a “dynamic centre” that “arises” from load perturbation and movement. From this perspective, it can be suggested that one role of the descending commands is to initiate an asymmetrical (left-right) and unbalanced (flexor-extensor) activation of the spinal cord system.

Such a relationship between loading and hip joint motion possibly occurs early in the development of gait. Indeed, in human babies [74] and lower mammals [75,76], the duration of the stance phase and its associated extensor muscle activity has been found to depend on both the position of the hip joint and the load borne by the standing leg.

## Conclusions

In conclusion, our findings suggest that after a facilitation of the NWR in the hip and knee flexors by descending motor commands, the sudden leg unloading and hip joint motion work in concert to produce an alternating (right and left) and crossed (flexors and extensors) reflex activity, possibly predisposing the legs to the cyclical pattern of walking.

## Abbreviations

NWR, Nociceptive withdrawal reflex; Gmax, Gluteus maximus; RF, Rectus femoris; VM, Vastus medialis; BF, Biceps femoris; TA, Tibialis anterior; SOL, Soleus.

## Competing interests

Financial competing interests

· Authors declare that, in the past five years, they have not received reimbursements, fees, funding, or salary from an organization that may in any way gain or lose financially from the publication of this manuscript, either now or in the future. No organization is financing this manuscript (including the article-processing charge). do not hold any stocks or shares in an organization that may in any way gain or lose financially from

· Authors declare that they the publication of this manuscript, either now or in the future.

· Authors declare that they do not hold or are currently applying for any patents relating to the content of the manuscript. They have not received reimbursements, fees, funding, or salary from an organization that holds or has applied for patents relating to the content of the manuscript.

· Authors declare that they have not other financial competing interests If so, please specify.

Non-financial competing interests

· Authors have no non-financial competing interests (political, personal, religious, ideological, academic, intellectual, commercial or any other) to declare in relation to this manuscript.

## Authors’ contributions

The work presented here was carried out in collaboration by all the authors. MS, AR, GS and FP defined the research theme. MS and AR designed the methods and experiments, carried out the laboratory experiments, analysed the data, interpreted the results and wrote the paper. OKA, CC, RD and SM co-designed the experiments and co-worked on associated data collection and interpretation. FD, FC and LP co-designed the experiments, and discussed the analyses, interpretation, and presentation. All the authors have contributed to, seen and approved the manuscript.
